# Impaired balance between coagulation and fibrinolysis plays a prominent role in patients with sepsis

**DOI:** 10.1186/cc13396

**Published:** 2014-03-17

**Authors:** Y Umemura, K Yamakawa, T Kiguchi, H Ogura, T Shimazu, S Fujimi

**Affiliations:** 1 Osaka General Medical Center, Osaka, Japan; 2 Osaka University Graduate School of Medicine, Osaka, Japan

## Introduction

The balance between coagulation and fibrinolysis was a prominent factor in the pathophysiology of sepsis, but this mechanism has been poorly understood. We aimed to determine whether collapsing this balance during the first day of sepsis correlates with progression of organ dysfunction and subsequent death.

## Methods

This study included all patients with sepsis admitted to a tertiary referral hospital in Japan. Global coagulation tests and hemostatic molecular markers such as fibrin/fibrinogen degradation products (FDP), D-dimer, thrombin-antithrombin complex (TAT) and plasmin-alpha 2 plasmin inhibitor complex (PIC) were measured within 12 hours after admission, and then SOFA and APACHE II scores and in-hospital mortality were evaluated. Patients were divided into three groups based on the levels of TAT/PIC and FDP/D-dimer and differences of clinical outcome between groups were assessed by chi-square analysis and ANOVA.

## Results

**Figure 1:**
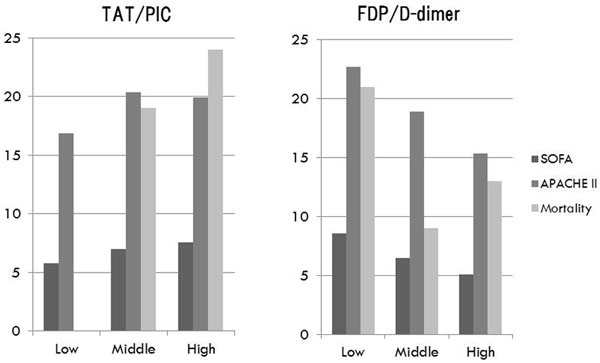
**Comparison of the characteristics between the three groups**.

We enrolled 101 patients; 87 patients survived, and 14 died. Mortality was significantly higher in the high TAT/PIC group (0%, 19% and 24% for low, middle and high TAT/PIC groups, respectively; *P *= 0.011). In addition, SOFA and APACHE II scores were significantly higher in the low FDP/D-dimer group (APACHE II = 22.3, 18.9 and 15.3; *P *< 0.01, SOFA = 8.6, 6.5 and 5.1; *P *< 0.01, for low, middle and high FDP/ D-dimer groups, respectively). See Figure 1.

## Conclusion

We demonstrated that the balance between coagulation and fibrinolysis, assessed with FDP/D-dimer and TAT/PIC ratios, was correlated with disease severity and clinical outcomes in sepsis, suggesting that impaired balance ofthe hemostatic system might play a pivotal role in progression of sepsis pathophysiology.

